# Engagement in cardiac rehabilitation after a first myocardial infarction: a qualitative evidence synthesis of patient experiences

**DOI:** 10.1080/17482631.2026.2693392

**Published:** 2026-07-21

**Authors:** Ziyuan Niu, Jie Zhao, Zhaoyang Ma, Yanan Sui, Li Xu

**Affiliations:** a School of Nursing, Shandong First Medical University, Jinan, People's Republic of China; b School of Nursing, Shandong Second Medical University, Weifang, People's Republic of China; c Jinan Central Hospital Affiliated to Shandong First Medical University, Jinan, People's Republic of China

**Keywords:** Myocardial infarction, cardiac rehabilitation, qualitative research, qualitative synthesis, patient engagement

## Abstract

**Purpose:**

To synthesize qualitative evidence on factors influencing patients’ participation in cardiac rehabilitation after a first acute myocardial infarction.

**Methods:**

A search of eight databases was conducted from inception to May 2026. Included studies were quality assessed using JBI criteria, and findings were synthesized using a hybrid approach that combined meta-aggregation for data extraction with thematic synthesis for interpretive analysis.

**Results:**

The synthesis of 9 qualitative studies yielded 33 synthesized findings. These findings were organized into three key themes: (1) the complex process of identity transformation following a first acute myocardial infarction (2) the dynamic interaction between motivators and barriers affecting participation in cardiac rehabilitation and (3) the fundamental importance of support systems in shaping patient experiences.

**Conclusion:**

Participation in cardiac rehabilitation among patients experiencing their first acute myocardial infarction is influenced by multiple factors. To enhance engagement, clinical healthcare providers should address identity disruption, strengthen intrinsic motivation, and facilitate peer and family support through tailored, multidisciplinary, and psychosocially integrated strategies.

## Graphical abstract

**Figure:**
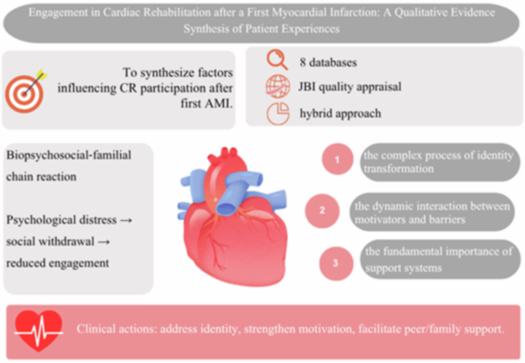

## Background

Acute myocardial infarction ranks among the leading causes of death from cardiovascular disease. Globally, over 7 million people suffer acute myocardial infarction annually, with a one-year mortality rate of approximately 10% among patients with a first acute myocardial infarction. Among survivors, 20% will experience another cardiovascular event within the first year (Piepoli et al., 2017). Data indicate that post-acute myocardial infarction prevention is crucial for reducing risk and adverse outcomes (Brouwers et al., 2020), making active cardiac rehabilitation essential for preventing recurrence and improving quality of life in first acute myocardial infarction patients. Cardiac rehabilitation (CR), as a comprehensive intervention, serves as a key strategy for reducing cardiovascular mortality and readmission rates (Ruivo et al., 2023). Its core encompasses multidimensional integrated rehabilitation approaches, including exercise guidance, medication management, and health education, aimed at improving cardiac function and enhancing patient quality of life. CR demonstrates significant efficacy in improving patient outcomes. Studies indicate that patients participating in cardiac rehabilitation programs experience a 20-25% reduction in mortality and a 30% decrease in readmission rates (Hansen et al., 2023). Recent quantitative studies have further demonstrated the benefits of cardiac rehabilitation on quality of life, depressive symptoms, and physical activity (Saleh et al., 2023; Saleh et al., 2025; Subih et al., 2024). Given its extensive benefits, the European Society of Cardiology (ESC) classifies cardiac rehabilitation as a highest-level recommended intervention for treating various cardiac conditions (Visseren et al., 2021). Despite near-universal endorsement of clinical guidelines, global cardiac rehabilitation uptake and completion rates remain low (Taylor et al., 2022). In developed countries, cardiac rehabilitation has become a standard component of cardiovascular disease treatment. However, in developing nations like China, its adoption remains extremely low and faces numerous challenges. These include insufficient public and healthcare provider awareness, uneven distribution of rehabilitation resources, limited health insurance support, and poor patient adherence (Corrado & Zorzi, 2024). Particularly for patients experiencing their first acute myocardial infarction—individuals undergoing disruption of their health equilibrium—their unique rehabilitation experience remains systematically unexplored and poorly understood. Therefore, this qualitative synthesis integrates existing qualitative research findings and the direct experiences of this population to comprehensively describe the experience of acute myocardial infarction patients participating in cardiac rehabilitation following their first episode. The aim is to provide insights for improving patient prognosis and quality of life.

## Design and methods

### Literature inclusion and exclusion criteria

We established inclusion and exclusion criteria using PICoS (Eriksen & Frandsen, 2018), where “Population” refers to patients with their first acute myocardial infarction, “Phenomenon of Interest” refers to cardiac rehabilitation experience, “Context” refers to hospital, community, or rehabilitation centre, and “Study type” refers to qualitative studies. Inclusion criteria: 1) Patients with first acute myocardial infarction; 2) Study focus: cardiac rehabilitation experience; 3) Study setting: hospital, community, or rehabilitation centre; 4) Study type: Qualitative or a qualitative component of mixed-method studies, including grounded theory, phenomenology, and descriptive studies. Exclusion criteria: Duplicate publications, incomplete data, inaccessible full texts, language other than English or Chinese, and conference proceedings. Reporting followed the PRISMA checklist (Page et al., 2021). (See Checklist, Supplemental material 2, for the completed checklist.) We reported synthesis findings following the ENTREQ framework. (See Checklist, Supplemental material 3, for the completed checklist.) The study is registered on PROSPERO (CRD420251172318).

### Literature search strategies

Searches were conducted in PubMed, Web of Science, Scopus, Embase, The Cochrane Library, China National Knowledge Infrastructure (CNKI), WANFANG Database, and VIP. The search period spanned from the inception of each database to May 2026. Searches employed a combination of subject headings and free-text terms, adjusted according to the characteristics of each database. Search terms included myocardial infarction, heart attack, myocardial infarct, heart infarction, cardiovascular stroke, cardiac rehabilitation, cardiovascular rehabilitation, perspective, lived experience, subjective experience, interview*, qualitative research, qualitative study, qualitative methodology, and grounded theory. The systematic search strategy is provided separately (see Document, Supplemental material 1).

### Literature screening and data extraction

Two researchers trained in evidence-based practice methods independently screened the literature according to strict inclusion and exclusion criteria and cross-checked the results. Data extraction included authors, countries, research methods, study subjects, phenomena of interest, and main findings. Data synthesis was conducted manually without the use of specialised software. Any disagreements during screening were resolved through discussion with a third researcher.

### Criteria for evaluating the methodological quality of literature

The quality of included qualitative studies was assessed using the Joanna Briggs Institute (JBI) Critical Appraisal Checklist for Qualitative Research (Lockwood et al., 2015). The checklist contains 10 criteria, each rated as “Yes,” “No,” or “Unclear.” Study quality was categorised as A (fully met criteria), B (partially met), or C (not met). Two researchers independently assessed the studies. Any disagreements were discussed with a third researcher. Table II presents the quality assessment results (the ten criteria are described in the table notes). During the screening and quality assessment stages, consensus was reached through discussion; therefore, a formal kappa statistic was not calculated.

### Literature analysis methodology

We used a hybrid approach combining JBI meta-aggregation (Lockwood et al., 2015) and thematic synthesis (Thomas & Harden, 2008). Structured aggregation using JBI meta-aggregation was used for data extraction and category formation, ensuring fidelity to the original findings of primary studies. Interpretive analysis using thematic synthesis was then employed to generate analytical themes that capture the dynamic relationships among the descriptive themes.

## Results

### Study inclusion

**Figure 1.**
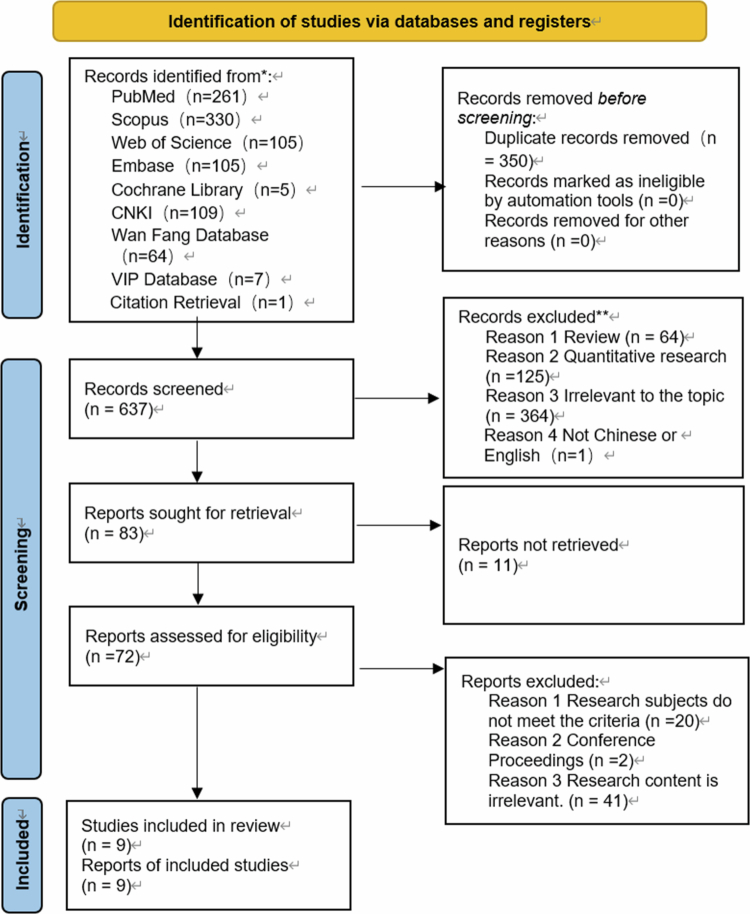
shows the PRISMA flow diagram of the study selection process for the qualitative evidence synthesis on cardiac rehabilitation engagement after the first acute myocardial infarction.

A preliminary search yielded 987 documents. After deduplication, screening, and literature evaluation, 9 documents were ultimately selected. The literature screening flowchart is shown in Figure 1.

### Basic characteristics and quality assessment results of included studies

The 9 included studies involved a total of 176 patients. Research methods varied, encompassing descriptive qualitative studies, phenomenology, interpretive phenomenology, grounded theory, and others. The basic characteristics of the included studies are presented in Table I, and the results of the methodological quality assessment are shown in Table II. The unmet needs of specific patient groups are presented in Table III.

**Table I.** Basic characteristics of the included literature.

Author Country	Years of inclusion of patients	Methods	Demographics (*n*, M/F)	Research Focus	Findings
Xu L. et al., China	2021-2022	Descriptive phenomenology	*n* = 29(25/4)	Motivation for Participating in Post-PCI Cardiac Rehabilitation Among AMI Patients	Three themes: The patient's physical experience, the patient's psychological state, social support
Zhu C. et al., USA	2022	Phenomenology	*n* = 18(0/18)	Young Female Patients with AMI: Perceptions of the Long-Term Rehabilitation Process	Eight themes: Loss of Security, Erosion of Self-Worth, Absence of Social Roles, Dual Losses of Hope and Optimism, Building Interpersonal Connections, Emotional Management Strategies, Meaning of Life, and Goal Setting
Wieslander I. et al., Sweden	2010	Descriptive phenomenology	*n* = 26(0/26)	A Dual Perspective on Preventive and Promotive Rehabilitation for Women with AMI	Three dimensions: behavioural, social, psychological
Simonÿ C.P. et al., Denmark	2012-2013	Interpretative phenomenology	*n* = 11(9/2)	Experiences of mild patients regarding their living conditions during cardiac rehabilitation	Three themes: Difficulty accepting the disease, understanding that life has become frail, An altered life
Hutton J.M. et al., UK	NR	Interpretative phenomenology	*n* = 10(10/0)	Male Patients' Experiences with MI and CR	Five themes: Making sense of the event, cognitive and emotional responses. Relationships with others, coping strategies, Experiences of health care
Hogg N.M. et al., UK	NR	Interpretative phenomenology	*n* = 6(6/0)	Personal Experiences and Meaning of Middle-Aged Male Patients with AMI in Coping with Their Illness	Three themes: Difficulty in Reflecting, Needing to Talk, Back to Normal
East L. et al., UK	NR	Descriptive phenomenology	*n* = 20(14/6)	The Experience of AMI Patients and Their Perspectives on CR Services	Four themes: the story of the heart attack, the way survivors’ lives have changed, experiences of existing services, and ideas for service development
Dreyer R.P. et al., USA	2016-2019	Participatory action research and phenomenology	*n* = 42(19/23)	Patients' Perspectives on AMI and Their Rehabilitation Experiences During Treatment	One core theme and two aspects: Identity Transformation, and Loss Aspects (Sense of Security, Social Roles, and Independence) and Gain Aspects (Emotional Presence, Connection, Purpose, and Hope).
Dilla D. et al., UK	2015-2016	Grounded theory	*n* = 14(10/4)	Experiences of South Asian Patients in Selecting and Implementing Lifestyle Changes During CR	Three themes: patronage of the family, conforming to beliefs, affinity towards one's group

Note: NR = not reported in the original study.

**Table II.** Evaluation of methodological quality of included studies.

Author	Item1	Item2	Item3	Item4	Item5	Item6	Item7	Item8	Item9	Item10	Overall Appraisal
Xu L. et al., China	Y	Y	Y	Y	Y	N	N	Y	Y	Y	B
Zhu C. et al., USA	Y	Y	Y	Y	Y	N	N	Y	Y	Y	B
Wieslander I. et al., Sweden	Y	Y	Y	Y	Y	N	N	Y	Y	Y	B
Simonÿ C.P. et al., Denmark	Y	Y	Y	Y	Y	N	N	Y	Y	Y	B
Hutton J.M. et al., UK	Y	Y	Y	Y	Y	N	N	Y	U	Y	B
Hogg N.M. et al., UK	Y	Y	Y	Y	Y	N	Y	Y	Y	Y	B
East L. et al., UK	Y	Y	Y	Y	Y	N	N	Y	U	Y	B
Dreyer R.P. et al., USA	Y	Y	Y	Y	Y	Y	Y	Y	Y	Y	A
Dilla D. et al., UK	Y	Y	Y	Y	Y	Y	Y	Y	Y	Y	A

Note: Items 1 to 10 correspond to the following criteria of the JBI checklist: (1) congruence between philosophical perspective and research methodology; (2) congruence between research methodology and research question; (3) congruence between methodology and data collection methods; (4) congruence between methodology and data analysis; (5) congruence between methodology and interpretation of results; (6) adequate locating of the researcher culturally or theoretically; (7) addressing the influence of the researcher; (8) adequate representation of participants and their voices; (9) ethical approval by an appropriate body; (10) conclusions drawn from the data analysis. Y = Yes (criterion met); N = No (criterion not met); U = Unclear. Overall appraisal grade: A = High quality (all or most criteria met); B = Moderate quality (some criteria not met); C = Low quality (few criteria met).

**Table III.** Unmet needs in specific patient subgroups.

Patient Subgroup	Identified Unmet Needs
Women	Symptoms overlooked, concerns dismissed, conflict in the caregiver role
Low socioeconomic status	Financial constraints, transportation challenges
Socially isolated patients	Lack of peer support, limited family support
Patients with low self-efficacy	Fear of exercise, loss of identity, lack of confidence

### Synthesis findings

From the included studies, we derived 40 initial findings. Through iterative synthesis, these were consolidated into 33 synthesised findings, which were then grouped into three main themes: (1) the complex process of identity transformation following a first acute myocardial infarction, (2) the dynamic interaction between motivators and barriers affecting participation in cardiac rehabilitation, and (3) the fundamental importance of support systems in shaping patient experiences. The integrated findings revealed that the rehabilitation experience for patients with a first acute myocardial infarction centred on a search for meaning amidst significant losses. We found that the process involved the transition from the “old self” to the “new self,” a shift facilitated by social supports and personal beliefs that enabled patients to rebuild their lives with a new perspective.

### Foundational barriers arising from the initial crisis

The initial crisis after a first acute myocardial infarction creates profound psychological and social barriers that directly undermine engagement in cardiac rehabilitation. Initially, symptom misinterpretation and diagnosis denial delay critical help-seeking: “I had pains in my left shoulder...I just thought that it was due to the lifting activity” (Simonÿ et al., 2017). These same factors undermine patients’ understanding of cardiac rehabilitation, hindering their later engagement. These misunderstandings, together with a loss of life motivation, directly erode the drive to participate in recovery, as illustrated by patients’ statements: “Everything seems completely pointless” (Hogg et al., 2007) and “I’m just lucky to be alive...everything happens for a reason…” (Dreyer et al., 2021). Simultaneously, overwhelming anxiety about safety during routine activities fosters a fear-driven avoidance of physical exertion, creating a direct behavioural impediment to prescribed exercise training. Beyond individual psychology, the identity shift to feeling like “a totally different person” or “an old man with a dickie heart” leads to shame and social retreat. One patient explained: “I always thought of myself as relatively young and healthy, and I feel like I’m an old man with a dickie heart” (Hutton & Perkins, 2008). This withdrawal diminishes access to crucial social support needed for sustained rehabilitation participation. Furthermore, the tension between returning to work and resisting a “weak” patient identity creates practical and psychological conflict. As one patient stated: “you can’t be a sculptor and not lift anything” (Hogg et al., 2007). Female patients often reported that their symptoms were invisible or dismissed by healthcare providers, highlighting a systemic failure to recognise their specific needs. A female patient noted, “Everybody hears about the men having the heart attacks and the chest pain, but women don’t talk about anything” (Zhu et al., 2025), potentially excluding them from tailored rehabilitation support. The initial experience itself is the very source of motivational deficits, behavioural avoidance, and social disconnection—all of which actively hinder rehabilitation.

### Intrinsic motivation

Patients' sustained participation was driven by intrinsic motivation—specifically, the tangible benefits they experienced from rehabilitation—which created a positive feedback loop that reinforced self-management. Crucially, rehabilitation not only boosted physical confidence (Li et al., 2024) but, through structured education, empowered patients to challenge themselves. One participant highlighted how this knowledge spurred personal growth: “Well, for example, the heart school taught me a lot and gave me the opportunity to take up gymnastics, which did so much for me. Otherwise I wouldn’t have dared to push myself so much” (Wieslander et al., 2016). When patients personally witness the positive transformations brought by rehabilitation, their motivation shifts from external demands to an intrinsic, sustainable driving force.

### External motivation

External motivators also played a significant role. Some patients applied a professional mindset from their occupations to rehabilitation, viewing it as a worthwhile investment and approaching challenges with comparative confidence: “This is equivalent to an investment…” and “How could this rehabilitation be harder than flying a plane?!” (Li et al., 2024). For others, family caregiving responsibilities provided a compelling reason for recovery: “I need to get myself back on my feet first before I can take care of my bedridden mother” (Li et al., 2024). This internally driven motivation, stemming from external factors, demonstrates that when rehabilitation behaviours are closely tied to cherished social identities and responsibilities, they can unleash powerful and sustainable momentum.

### Emotional burden

Patients faced significant emotional barriers. Pessimism and a sense of being overwhelmed were common, particularly when managing multiple health issues. One patient stated, “I have to undergo haemodialyses regularly, so I can't manage this” (Li et al., 2024). Anxiety was also prevalent. Some patients feared a relapse, as one participant stated: “I worry if I’m going to have another one... it makes you feel nervous” (East et al., 2004). Among young women, the stress of balancing heart health with life's demands added another layer: “It’s just really overwhelming…” (Zhu et al., 2025). In severe cases, this could lead to feelings of despair and futility. Collectively, these negative emotions eroded patients' willingness to participate in rehabilitation, creating a vicious cycle where fear inhibited action.

### Risk perception

Without professional guidance, patients commonly harbour severe misconceptions and fears regarding cardiac rehabilitation, particularly exercise training: “I'm afraid the bracket will fall out” (Li et al., 2024). While some patients recognise that dietary adjustments and habit changes—such as quitting smoking or drinking—can effectively improve their condition, increasing exercise volume remains a significant challenge: “I don’t think I will now…unless I do something stupid like go outside and start doing too much” (East et al., 2004). This fundamental cognitive dissonance towards exercise severely undermined adherence.

### Real-world burden

Practical socioeconomic circumstances were pivotal. Financial constraints directly forced some to abandon rehabilitation to return to work or due to prohibitive costs: “The bills can’t get paid without me working…” (Zhu et al., 2025). Employment-related conflicts also created barriers, from concerns about requesting leave in a new job to the simple difficulty of scheduling sessions around work. Additionally, gendered role expectations added a layer of complexity, as female patients reported deprioritizing their own symptoms amidst work and family duties: “Being a woman, a working woman who will ignore physical pain…” (Zhu et al., 2025).

### Social support and family roles

Social support served as a crucial facilitator. For some, family responsibilities were internalised as a positive motivator, particularly for young women striving to maintain their caregiver role through recovery: “Everything else can be on hold, but when it comes to the children and the food… that’s what you think about” (Zhu et al., 2025). Family support often evolved into a shared emotional endeavour, transforming personal goals into collective family targets and providing essential encouragement: “I don’t do it for myself, I do it for them (family)” (Dilla et al., 2020). In addition to family, peer support also played a distinct and complementary role, interacting with others who shared the same diagnosis fostered motivation: “He's recovering so well, it's giving me motivation” (Li et al., 2024) and a sense of community (Wieslander et al., 2016). Moreover, peers offered a unique safe space to express vulnerabilities that patients felt unable to share with family, for fear of worrying them (East et al., 2004).

### The desire for and experience of professional support

Patients emphasised that effective professional support must provide clear, actionable guidance; vague advice left them feeling at a loss (Li et al., 2024). The specialised expertise of the rehabilitation team was foundational, generating a crucial sense of security that enabled patients to engage confidently. Beyond competence, the encouraging attitude of professionals served as a vital motivator for long-term participation. As one patient noted, direct encouragement from a doctor was transformative: “If you change your ways…you’ll stand more of a chance of it not happening to you. So that’s just made me change the best I can” (East et al., 2004).

## Discussion

The Diversity and Complexity of Cardiac Rehabilitation Experiences Among Patients with First Acute Myocardial Infarction.

We found that the participation experience in cardiac rehabilitation for patients with a first acute myocardial infarction is a complex, dynamic process encompassing multiple dimensions such as disease cognition, psychological responses, social roles, and rehabilitation practices. Crucially, these elements are not isolated but interdependent, forming a chain reaction involving physiological, psychological, social, and familial roles. Research indicates (Drory et al., 1999; Li et al., 2022) that newly diagnosed patients often experience a state of disorientation. Among them, patients with weaker psychological coping abilities exhibit a more pronounced sense of disease uncontrollability. This feeling of loss of control not only leads to emotional burdens such as anxiety and fear but also further triggers a shift in identity and a degree of social withdrawal. The identity shift from the “old self” to the “new self” observed in our findings aligns with the concept of biographical disruption (Bury, 1982). This concept describes how a major health event fractures one's taken-for-granted life trajectory, necessitating a reconstruction of self-identity. This process also reflects the loss and rebuilding of the self-described in the chronic illness literature (Charmaz, 1983). Unlike previous qualitative syntheses that listed barriers as independent factors (Neubeck et al., 2012), our findings reveal a order in which psychological and social factors influence each other. We term this the biopsychosocial‑familial chain reaction: physiological threat leads to psychological distress, which then upsets social roles and family dynamics, ultimately undermining rehabilitation engagement.

We also identified distinct challenges related to social roles and resources. Gender disparities were particularly clear: female patients often faced more severe obstacles. Beyond disease management, their rehabilitation is compounded by gendered expectations and the accumulation of caregiving and career responsibilities, aligning with literature on the caregiver-health conflict for women (Martinez-Marcos & De la Cuesta-Benjumea, 2015; Tavero et al., 2018). Additionally, occupational and economic status are not merely background but key determinants of participation, influencing motivation, options, and adherence—confirming socioeconomic status as a critical factor in chronic disease management (Katikireddi et al., 2017; Lawless et al., 2025). Recent quantitative studies reflect that these social and economic challenges are compounded by psychological mechanisms. For instance, Saleh et al. (2023) identified predictors of physical activity behaviour change; our synthesis reveals that psychological factors such as loss of control and identity disruption may underlie those predictors. Similarly, the improvement in depressive symptoms reported by Saleh et al. (2025) with home‑based rehabilitation suggests the importance of addressing psychological mechanisms.

The study found that identity disruption, lack of motivation, and insufficient support may have persistent long-term effects. If identity disruption persists over time, patients may continue to avoid exercise and medical follow-ups, leading to reduced adherence and an increased risk of rehospitalization and relapse (Charmaz, 1983). Conversely, sustained intrinsic motivation can drive patients to autonomously maintain healthy behaviours, which is associated with lower all-cause mortality. Furthermore, strong social support provides ongoing emotional buffering and behavioural reinforcement. Studies show that patients with good social support have significantly lower mortality and reinfarction rates following myocardial infarction (Mookadam & Arthur, 2004). Therefore, addressing these factors is not only crucial for initial rehabilitation participation but also key to improving long-term clinical outcomes.

From Guidelines to Practice: Providing Holistic Support for the Rehabilitation Process CR is defined as a comprehensive, personalised intervention (Ambrosetti et al., 2021), yet its implementation is suboptimal due to low participation (Benzer et al., 2017). This meta-synthesis reveals the root cause: the biopsychosocial-familial chain reaction remains unaddressed. Single-dimensional interventions fail to break this cycle. Therefore, moving from a biomedical model to holistic, patient-centred care is essential. This requires rehabilitation teams to proactively identify and address the specific physiological, psychological, or social barriers hindering each patient, ensuring support precisely meets their needs to enhance self-efficacy. Based on our findings, we recommend the following strategies: First, conduct routine psychosocial screening during early rehabilitation visits to identify patients at risk of maladaptive perceptions (e.g. fear of exercise, perceived fragility) and emotional distress (e.g. anxiety, withdrawal). Second, offer tailored psychological support based on screening results. For patients with fear-driven avoidance, provide clear, individualised education on exercise safety and symptom management. For those experiencing identity loss or social withdrawal, consider referral to peer support programs or counselling services. Third, facilitate family and peer involvement by providing structured guidance to family caregivers on how to offer practical and emotional support without reinforcing patient dependency. Peer support groups, either in-person or virtual, can help patients share experiences and rebuild confidence. Fourth, address socioeconomic barriers by connecting patients with financial counselling, transportation assistance, or home-based rehabilitation when centre-based programs are not feasible. Our findings also support the use of home-based or digitally assisted rehabilitation, which evidence suggests can address fear of movement and logistical barriers (Taylor et al., 2022).

These strategies can help clinicians translate guidelines into practice and empower patients to achieve their rehabilitation goals.

## Conclusion

This study employed a hybrid qualitative evidence synthesis to systematically review 9 qualitative studies, exploring the experiences of patients with their first acute myocardial infarction in cardiac rehabilitation. It also explored the factors influencing patients' participation in cardiac rehabilitation and their enhanced self-efficacy for rehabilitation. The integrated findings suggest that participation in cardiac rehabilitation among first acute myocardial infarction patients is influenced by multiple factors across various dimensions. Overall, by mapping the biopsychosocial-familial chain reaction, this synthesis offers an integrated framework that extends previous barrier lists and provides a conceptual basis for the multifaceted, stepwise intervention strategies outlined in the Discussion. Future efforts should bridge the gap between patients' actual experiences and ideal guidelines by implementing tailored, multidisciplinary, and psychosocially integrated strategies to enhance the effectiveness and adherence of cardiac rehabilitation.

This study also has several limitations: Differences in age and socioeconomic background across included studies, as well as variations in healthcare systems, rehabilitation programs, and cultural contexts across countries, may have impacted the transferability of the findings. Given the limited number of studies per country, a systematic cross-country comparison was not feasible; however, future research should explore how these contextual factors shape patient experiences. Furthermore, because the included studies varied in their gender composition—with some involving only women, others only men, and others both—a systematic gender-based analysis was not feasible within the scope of this review. Future research could explore gender-specific experiences in cardiac rehabilitation. Additionally, while the included studies focused on patients' rehabilitation experiences and perceptions, future research could also analyse perspectives from healthcare providers and rehabilitation teams to inform the development of more comprehensive and effective cardiac rehabilitation strategies.

## Supplementary Material

supplementary material1_SearchStrategy.pdf supplementary material1_SearchStrategy.pdf

supplementary material2_PRISMA_Checklist.pdf supplementary material2_PRISMA_Checklist.pdf

supplementary material3_ENTREQ_Checklist.pdf supplementary material3_ENTREQ_Checklist.pdf

## Data Availability

The authors confirm that the data supporting the findings of this study are available within the article and its supplementary materials.
