# Supplementary material for: Engagement in cardiac rehabilitation after a first myocardial infarction: a qualitative evidence synthesis of patient experiences
Source: Int J Qual Stud Health Well-being. 2026 Jul 21;21(1):2693392. doi: 10.1080/17482631.2026.2693392 (PMC13390156; doi:10.1080/17482631.2026.2693392)
Supplement: supplementary material1_SearchStrategy.pdf [file ZQHW_A_2693392_SM1964.pdf]

Supplementary material 1 (Full Search Strategy)

Database: Pubmed

Total 262

#2	Search: ((“myocardial infarction”[Title/Abstract] OR “heart attack”[Title/Abstract] OR “myocardial infarct*”[Title/Abstract] OR “heart infarction”[Title/Abstract] OR “cardiovascular stroke”[Title/Abstract]) AND (“cardiac rehabilitation”[Title/Abstract] OR “cardiovascular rehabilitation”[Title/Abstract])) AND (perspective[Title/Abstract] OR “lived experience”[Title/Abstract] OR experience[Title/Abstract] OR “subjective experience”[Title/Abstract] OR interview[Title/Abstract] OR “grounded theory”[Title/Abstract] OR “qualitative research”[Title/Abstract] OR “qualitative study”[Title/Abstract] OR “qualitative methodology”[Title/Abstract] OR “phenomenological research”[Title/Abstract])	252
#5	Search: (((“Myocardial Infarction”[Mesh]) AND “Cardiac Rehabilitation”[Mesh]) AND “Qualitative Research”[Mesh]	12
#6	Search: (((“Myocardial Infarction”[Mesh]) AND “Cardiac Rehabilitation”[Mesh]) AND “Qualitative Research”[Mesh]) OR (((“myocardial infarction”[Title/Abstract] OR “heart attack”[Title/Abstract] OR “myocardial infarct*”[Title/Abstract] OR “heart infarction”[Title/Abstract] OR “cardiovascular stroke”[Title/Abstract]) AND (“cardiac rehabilitation”[Title/Abstract] OR “cardiovascular rehabilitation”[Title/Abstract])) AND (perspective[Title/Abstract] OR “lived experience”[Title/Abstract] OR experience[Title/Abstract] OR “subjective experience”[Title/Abstract] OR interview[Title/Abstract] OR “grounded theory”[Title/Abstract] OR “qualitative research”[Title/Abstract] OR “qualitative study”[Title/Abstract] OR “qualitative methodology”[Title/Abstract] OR “phenomenological research”[Title/Abstract]))	256
7	Search: (((“Myocardial Infarction”[Mesh]) AND “Cardiac Rehabilitation”[Mesh]) AND “Qualitative Research”[Mesh]) OR (((“myocardial infarction”[Title/Abstract] OR “heart attack”[Title/Abstract] OR “myocardial infarct*”[Title/Abstract] OR “heart infarction”[Title/Abstract] OR “cardiovascular stroke”[Title/Abstract]) AND (“cardiac rehabilitation”[Title/Abstract] OR “cardiovascular rehabilitation”[Title/Abstract])) AND (perspective[Title/Abstract] OR “lived experience”[Title/Abstract] OR experience[Title/Abstract] OR “subjective experience”[Title/Abstract] OR interview[Title/Abstract] OR “grounded theory”[Title/Abstract] OR “qualitative research”[Title/Abstract] OR “qualitative study”[Title/Abstract] OR “qualitative methodology”[Title/Abstract] OR “phenomenological research”[Title/Abstract])) AND ("2025/11/01"[Date - Publication] : "2026/05/15"[Date - Publication])	6

Database: Embase

Total 105

#1	"Heart infarction"/exp	529,957
#2	"acute infarct".ti,ab,kw OR "cardiac infarction".ti,ab,kw OR "acute myocardial infarction".ti,ab,kw OR	377,475

	"myocardial infarction".ti,ab,kw OR "heart attack".ti,ab,kw OR "Heart muscle infarction".ti,ab,kw	
#3	#1 OR #2	583,482
#4	"heart rehabilitation"/exp	20,629
#5	"cardiac rehabilitation".ti,ab,kw OR "cardiac rehabilitation unit".ti,ab,kw OR "coronary rehabilitation".ti,ab,kw OR "infarct rehabilitation".ti,ab,kw OR "Heart rehabilitation".ti,ab,kw	18,268
#7	#4 OR #5	23,841
#8	"qualitative research"/exp	159,010
#9	"qualitative studies".ti,ab,kw OR "qualitative study".ti,ab,kw OR "qualitative research".ti,ab,kw	142,992
#10	#8 OR #9	107,555
#11	#3 AND #7 AND #10	105

Database: Cochrane Central Register of Controlled Trials (CENTRAL)

Total 5

("myocardial infarction" OR "heart attack" OR "myocardial infarct" OR "heart infarction" OR "cardiovascular stroke") ti,ab,kw AND ("cardiac rehabilitation" OR "cardiovascular rehabilitation") ti,ab,kw AND (perspective OR "lived experience" OR experience OR "subjective experience" OR interview* OR "grounded theory" OR "qualitative research" OR "qualitative study" OR "qualitative methodology" OR "phenomenological research" OR "ethnographic research" OR "ethnographic study") ti,ab,kw

Database: Web of Science

Total 105

TS= ("myocardial infarction" OR "heart attack" OR "myocardial infarct*" OR "heart infarction" OR "cardiovascular stroke") AND TS= ("cardiac rehabilitation" OR "cardiovascular rehabilitation") AND TS= (interview* OR "qualitative research" OR "qualitative methodology" OR "phenomenological research" OR "ethnographic research") AND TS= ("lived experience" OR experience OR "subjective experience")

Database: Scopus

Total 330

(TITLE-ABS-KEY ("myocardial infarction" OR "heart attack" OR "myocardial infarct*" OR "heart infarction" OR "cardiovascular stroke") AND TITLE-ABS-KEY ("cardiac rehabilitation" OR "cardiovascular rehabilitation") AND TITLE-ABS-KEY (interview* OR "grounded theory" OR "qualitative research" OR "qualitative study" OR "qualitative methodology" OR "phenomenological research" OR perspective OR "lived experience"))

Database: CNKI

Total 109

((Subject: Acute myocardial infarction) OR (Article abstract: Acute myocardial infarction + Acute myocardial infarction + Myocardial infarction))

AND

((Subject: Cardiac Rehabilitation) OR (Abstract Keywords: Cardiac Rehabilitation + Cardiovascular Rehabilitation + Comprehensive Cardiac Rehabilitation + Cardiac Rehabilitation Nursing))

AND

((Abstract Keywords: Qualitative Research + Qualitative Study + Phenomenology + Interview + Experience + Feelings + Perceptions + Views + Perspectives + Beliefs + Attitudes))

Database: VIP

Total 7

(Title or Keywords: Acute Myocardial Infarction OR Title or Keywords: Acute Myocardial Infarction OR Title or Keywords: Myocardial Infarction)

AND

(Title or Keywords: Cardiac Rehabilitation OR Title or Keywords: Cardiovascular Rehabilitation OR Title or Keywords: Comprehensive Cardiac Rehabilitation OR Title or Keywords: Cardiac Rehabilitation Nursing)

AND

(Title or Keywords: Qualitative research OR Title or Keywords: Qualitative study OR Title or Keywords: Experience OR Title or Keywords: Feelings OR Title or Keywords: Perceptions OR

Title or Keywords: Views OR Title or Keywords: Perspectives)

Database: WANFANG

Total 64

(Subject: Acute myocardial infarction OR Title or Keywords: Acute myocardial infarction OR Title or Keywords: Myocardial infarction)

AND

(Subject: Cardiac rehabilitation OR Title or Keywords: Cardiovascular rehabilitation OR Title or Keywords: Comprehensive cardiac rehabilitation OR Title or Keywords: Cardiac rehabilitation nursing)

AND

(Title or Keywords: Qualitative research OR Title or Keywords: Qualitative study OR Title or Keywords: Experience OR Title or Keywords: Feelings OR Title or Keywords: Perceptions OR Title or Keywords: Views OR Title or Keywords: Perspectives)
