# Supplementary material for: Engagement in cardiac rehabilitation after a first myocardial infarction: a qualitative evidence synthesis of patient experiences
Source: Int J Qual Stud Health Well-being. 2026 Jul 21;21(1):2693392. doi: 10.1080/17482631.2026.2693392 (PMC13390156; doi:10.1080/17482631.2026.2693392)
Supplement: supplementary material3_ENTREQ_Checklist.pdf [file ZQHW_A_2693392_SM1966.pdf]

The ENTREQ Checklist

**Item**	**Guide and description**	**Reported on page**
Aim	State the research question the synthesis addresses.	2
Synthesis methodology	Identify the synthesis methodology or theoretical framework which underpins the synthesis, and describe the rationale for choice of methodology *(e.g. meta-ethnography, thematic synthesis, critical interpretive synthesis, grounded theory synthesis, realist synthesis, meta-aggregation, meta-study, framework synthesis).*	3
Approach to searching	Indicate whether the search was pre-planned *(comprehensive search strategies to seek all available studies)* or iterative *(to seek all available concepts until they theoretical saturation is achieved).*	3
Inclusion criteria	Specify the inclusion/exclusion criteria *(e.g. in terms of population, language, year limits, type of publication, study type).*	3
Data sources	Describe the information sources used (e.g. *electronic databases (MEDLINE, EMBASE, CINAHL, psycINFO, Econlit) grey literature databases (digital thesis, policy reports), relevant organisational websites, experts, information specialists, generic web searches (Google Scholar) hand searching, reference lists)* and when the searches conducted; provide the rationale for using the data sources.	2-3
Electronic Search strategy	Describe the literature search *(e.g. provide electronic search strategies with population terms, clinical or health topic terms, experiential or social phenomena related terms, filters for qualitative research, and search limits).*	2-3
Study screening methods	Describe the process of study screening and sifting *(e.g. title, abstract and full text review, number of independent reviewers who screened studies).*	3
Study characteristics	Present the characteristics of the included studies *(e.g. year of publication, country, population, number of participants, data collection, methodology, analysis, research questions).*	Table 1
Study selection results	Identify the number of studies screened and provide reasons for study exclusion *(e.g. for comprehensive searching, provide numbers of studies screened and reasons for exclusion indicated in a figure/flowchart; for iterative searching describe reasons for study exclusion and inclusion based on modifications the research question and/or contribution to theory development).*	Figure 1
Rationale for appraisal	Describe the rationale and approach used to appraise the included studies or selected findings *(e.g. assessment of conduct (validity and robustness), assessment of reporting (transparency), assessment of content and utility of the findings).*	3
Appraisal items	State the tools, frameworks and criteria used to appraise the studies or selected findings (e.g. Existing tools: CASP, QARI, COREQ, Mays and Pope [25]; reviewer developed tools; describe the domains assessed: research team, study design, data analysis and interpretations, reporting).	3
Appraisal process	Indicate whether the appraisal was conducted independently by more than one reviewer and if consensus was required.	3
Appraisal results	Present results of the quality assessment and indicate which articles, if any, were weighted/excluded based on the assessment and give the rationale.	Table 2
Data extraction	Indicate which sections of the primary studies were analysed and how were the data extracted from the primary studies? *(e.g. all text under the headings "results /conclusions" were extracted*	3-4

	electronically and entered into a computer software).	
Software	State the computer software used, if any.	Not applicable
Number of reviewers	Identify who was involved in coding and analysis.	3
Coding	Describe the process for coding of *data (e.g. line by line coding to search for concepts).*	3
Study comparison	Describe how were comparisons made within and across studies *(e.g. subsequent studies were coded into pre-existing concepts, and new concepts were created when deemed necessary).*	3-4
Derivation of themes	Explain whether the process of deriving the themes or constructs was inductive or deductive.	3-4
Quotations	Provide quotations from the primary studies to illustrate themes/constructs, and identify whether the quotations were participant quotations of the author's interpretation.	4-7
Synthesis output	Present rich, compelling and useful results that go beyond a summary of the primary studies *(e.g. new interpretation, models of evidence, conceptual models, analytical framework, development of a new theory or construct).*	4-7
